# Genomic determinants of antifungal activity of *Streptomyces melanosporofaciens* STM-2 revealed by genome mining, comparative genomics and evolutionary analysis

**DOI:** 10.3389/fmicb.2026.1787011

**Published:** 2026-03-20

**Authors:** Muhammed Opoku Gyamfi, Ming-Jen Cheng, Jyh-Yih Leu, Min Tseng, Joshua Addo, Ai-Ling Hour, Wei-Chung Cheng

**Affiliations:** 1 Graduate Institute of Applied Science and Engineering, Fu Jen Catholic University, New Taipei City, Taiwan; 2 Department of Life Science, Fu Jen Catholic University, New Taipei City, Taiwan; 3 Department of Agricultural, Nutrition and Food Systems, University of New Hampshire, Durham, NH, United States

**Keywords:** AlphaFold2, antifungal activity, BGC, biocontrol, comparative genomics, genomics, STM-2, *Streptomyces*

## Abstract

**Introduction:**

*Streptomyces* species are prolific producers of bioactive compounds, and they play a role in biological control in agricultural systems.

**Materials and methods:**

In the current study, we employed an integrated approach that involved physiological and biochemical characterization, genome mining, comparative genomics, and evolutionary analysis to elucidate the specific genomic determinants underlying the antifungal activity exhibited by STM-2, a novel *Streptomyces melanosporofaciens* strain, isolated from Chiayi County, Taiwan.

**Results and discussion:**

Initial physiological and biochemical characterization, complemented by enzymatic screening, revealed the secretion of antifungal hydrolytic enzymes, including protease, CMCase, pectinase and xylanase, which actively play a role in fungal growth inhibition and fungal cell wall degradation. To elucidate the molecular basis of this activity, a wholegenome sequence was obtained and annotated, revealing a typical high-GC (71%) linear chromosome. Genome mining using antiSMASH predicted an extensive repertoire of 52 biosynthetic gene clusters (BGCs), encompassing type I and II PKS, NRPS, and hybrid PKS–NRPS systems, RiPPs, siderophores, terpenes, and diverse tailoring enzymes (P450s, glycosyltransferases, halogenase candidates). Again, we identified genes tentatively responsible for antifungal activity, including chitinases, β-1,3-glucanases, xylanses, pectinases, and proteases. Comparative pangenomic analysis, supported by high-resolution Average Nucleotide Identity (ANI) and digital DNA–DNA hybridization (dDDH) scores, revealed the evolutionary uniqueness of the strain, identifying an accessory genome containing unique genes specifically associated with specialized antifungal activity. Further in silico structural analysis of these unique gene products using AlphaFold2 yielded threedimensional protein models with very high pLDDT scores, providing high-confidence structural evidence for their specialized functional roles. Collectively, these results provide a comprehensive understanding of the genomic determinants required for potent biocontrol activity, positioning STM-2 as a promising candidate for biocontrol and further research in biotechnological applications.

## 1 Introduction

The genus *Streptomyces* comprises filamentous actinobacteria that play pivotal ecological roles in soil environments and are renowned for their capacity to produce a wide diversity of bioactive compounds and extracellular enzymes. Members of this genus account for a substantial proportion of clinically and agriculturally important antifungal agents, as well as enzymes involved in the degradation of complex biopolymers, contributing to microbial competition and biocontrol activity (Barka et al., 2016; Chater, 2016). Plant diseases caused by pathogenic microorganisms represent a substantial challenge to global agriculture, resulting in approximately 30–40% crop damage and significant economic losses annually (Khan et al., 2023). Although chemical fungicides are widely used, their prolonged application has led to the emergence of resistant phytopathogens and bioaccumulation of toxic residues, raising concerns about environmental sustainability. Thus, there is an urgent need to develop eco-friendly biological control agents (BCAs) (Xu et al., 2024), and members of the genus *Streptomyces* are among the most promising candidates due to their extensive secondary metabolism and biosynthetic potential. Despite extensive exploitation of *Streptomyces* for natural product discovery, the genomic determinants underlying strain-specific antifungal activity remain incompletely resolved.

The efficacy of *Streptomyces* as a BCA is attributed to multiple mechanisms, including the production of antifungal secondary metabolites such as polyketides, non-ribosomal peptides (NRPs), and ribosomally synthesized and post-translationally modified peptides (RiPPs), and the secretion of hydrolytic enzymes like chitinases, proteases, pectinases, and xylanases with some capable of degrading fungal cell walls (Khan et al., 2023). Whole-genome sequencing and comparative genomics have revealed that *Streptomyces* species possess large, open pan-genomes characterized by extensive accessory gene content, including biosynthetic gene clusters (BGCs), horizontally acquired genes, and lineage-specific adaptations (Doroghazi et al., 2014; McDonald and Currie, 2017). Comparative analyses have demonstrated that closely related strains often differ markedly in their antifungal phenotypes, largely due to variation in the accessory genome rather than the conserved core genome. Recently, integrative approaches combining pan-genome analysis, synteny mapping, and evolutionary reconstruction are increasingly employed to link genomic variation with functional traits. Earlier studies by Xu et al. (2024) and Gonzalez-Silva et al. (2024) reported the integration of comparative pangenomic analysis, genome mining and evolutionary analysis to reveal the genomic determinants of antimicrobials and biosynthetic potential of *Streptomyces* strains. The genetic mechanisms of many potent strains remain largely unexplored; it is then imperative to conduct comprehensive genomic characterizations to elucidate the genetic basis of antagonistic activity and uncover the molecular determinants required for sustainable biotechnological applications.

To bridge the gap between genomic prediction and functional understanding, the integration of artificial intelligence in structural biology has become a pivotal tool in recent years (Auslander et al., 2021). The development of AlphaFold2 has enabled the prediction of protein structures with near-experimental accuracy. The reliability of these models is quantified by the predicted local-distance difference test (pLDDT), where high scores indicate confident atomic-level accuracy in the predicted folds of enzymes and biosynthetic proteins (Auslander et al., 2021).

This research project characterizes a novel strain of *Streptomyces melanosporofaciens* (strain STM-2) showing potent growth inhibition against major plant pathogens. Earlier studies have reported the application of some *S. melanosporofaciens* strains as biocontrol agents (strain X216 reduced clubroot disease severity in *Brassica napus;* Ding et al., 2023; EF-76 inhibited growth of common scab of potato; Beauséjour et al., 2003); however, there was no thorough study detailing the genomic determinants of these traits in *S. melanosporofaciens.* Through an integrated approach that combines physiological characterization, genome mining, comparative genomics, and evolutionary analysis, we elucidate the specific genomic determinants, including unique BGCs and antifungal enzymes, that drive the biocontrol and biosynthetic potential of STM-2. High-confidence protein structure predictions were used to complement genomic findings by assessing the structural integrity of selected antifungal-associated proteins. Together, these analyses provide a comprehensive genomic and evolutionary perspective on the determinants of antifungal activity and biosynthetic potential in *Streptomyces melanosporofaciens* STM-2.

## 2 Materials and methods

### 2.1 Isolation of strain STM-2, morphological and cultural characteristics of strain STM-2

The strain STM-2 was isolated from the soil sample of Haomei Wetlands, Budai Township, Chiayi County, Taiwan (23.364132°, 120.130905°). The soil sample was dried at room temperature for 7 days, followed by suspending 2 g of the dried sample in 18 mL of sterile distilled water. The suspension was then subjected to shaking for 1 h and allowed to settle. Exactly 0.1 mL of the supernatant was plated on humic acid-vitamin (HV) agar (Hayakawa and Nonomura, 1987) supplemented with cycloheximide (50 mg /L) and nalidixic acid (20 mg/L). The cultured plates were incubated at 28 °C for 4 weeks. The strain STM-2 was purified and maintained on inorganic salt-starch agar slants and as suspensions of spores or mycelial fragments in glycerol (20%, v/v) stored at −20 °C.

Morphological characteristics of the strain STM-2 were observed by scanning electron microscopy (S-4700, Hitachi) after incubating the strain on inorganic salt-starch agar for 14 days at 28 °C and fixation in 4% osmium tetroxide solution. The sample was then dehydrated through a series of ethanol-acetone solutions and critical point dried. Cultural characteristics were evaluated using 21-day-old cultures grown at 28 °C on various media (ISP2, ISP3, ISP4, and ISP5). The ISCC-NBS Colour-Name Charts (Kelly, 1958) were used to determine the substrate mycelium’s color designations. Earlier procedures described by Shirling and Gottlieb (1966), Goodfellow (1971), and Gordon et al. (1974) were used to determine physiological and biochemical features and carbon source utilization. The qualitative assays for extracellular enzymes were carried out in inorganic starch solid media with pH of about 6.8–7.2 and incubated for 3–7 days at 26–30 °C. STM-2 was then screened for extracellular amylase (starch-iodine Test), protease (Milk/Casein agar), CMCase (Congo red technique), pectinase (pectin), and xylanase (xylan).

### 2.2 Molecular identification of strain STM-2 and phylogenetic analysis

**Figure 1:**
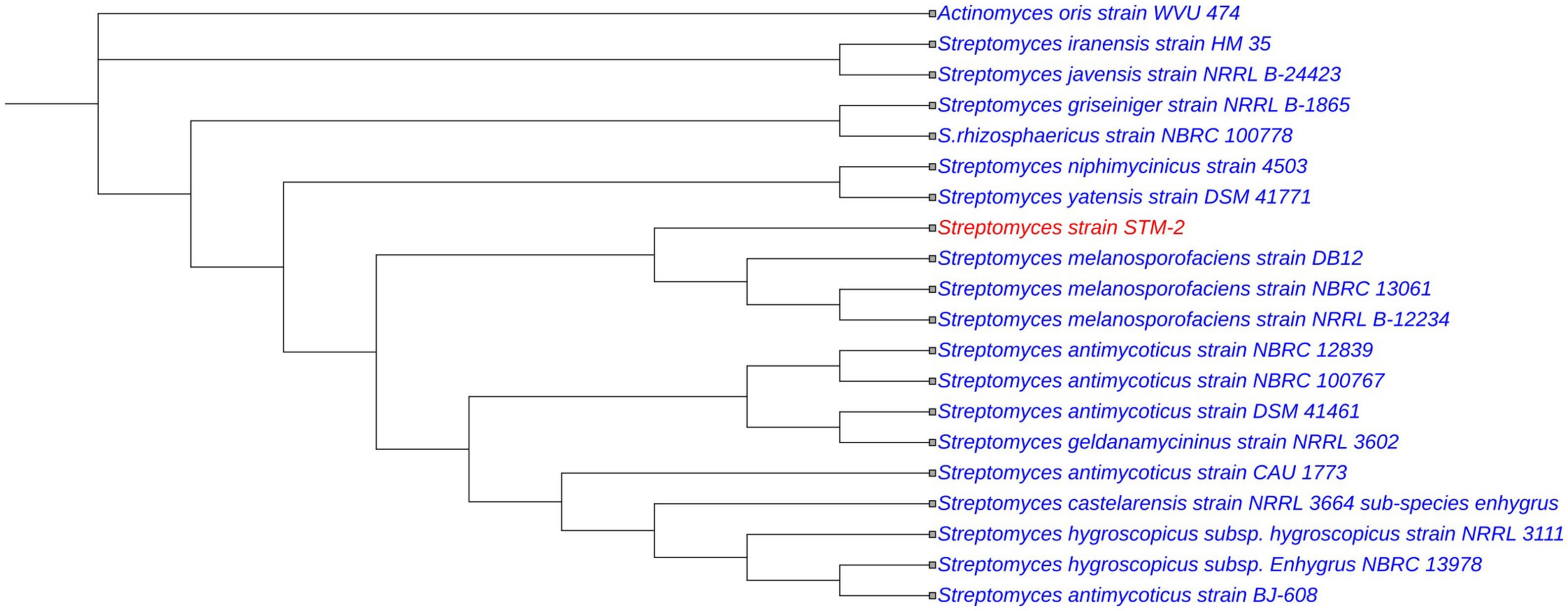
Neighbor-joining phylogenetic tree based on 16S rRNA gene sequences of strain STM-2 and closely related species within the genus *Streptomyces*, along with *Actinomyces oris* strain WVU474. Node numbers indicate bootstrap values, expressed as percentages from an analysis of 1,000 resampled datasets. The scale bar represents 0.02 substitutions per nucleotide position.

For DNA extraction and 16S rRNA gene sequencing, strain STM-2 was grown in TSB at 30 °C for 5 days. Cells were removed from the broth by using a pipette tip, and total DNA was extracted by using a Qiagen genomic DNA kit following the manufacturer’s protocol. The 16S rRNA gene amplification and sequencing were performed using the conserved primers 27F (5′-GAG TTT GAT CCT GGC TCA G-3′) and 1492R (5′-ACG GCT ACC TTG TTA CGA CTT-3′) (Gupta et al., 2014). The sequencing of the purified genomic DNA was done by Tri-I Biotech Inc. (Taipei, Taiwan). The 16S rRNA gene sequence was analyzed using the EzBioCloud database (http://eztaxon-e.ezbiocloud.net/; Chalita et al., 2024) and the NCBI BLAST GenBank 16S rRNA database to determine pairwise sequence similarities through global alignment algorithms. Closely related *Streptomyces* strains, along with one *Actinomyces oris* strain, were selected for phylogenetic analysis in MEGA 12.0 (Kumar et al., 2024) using the built-in Clustal_X algorithm. Phylogenetic trees were constructed by the neighbor-joining method (Figure 1), maximum-likelihood, and maximum-parsimony algorithms (Supplementary Figure S1). Kimura’s two-parameter model was used to compute the evolutionary distances for the neighbor-joining tree.

### 2.3 Determination of antagonistic activity of *Streptomyces* sp. strain STM-2 against phytopathogens

To evaluate the antagonistic effect of *Streptomyces* sp. strain STM-2, a dual culture assay was conducted against ten phytopathogenic fungi (Table 1) from different genera known to affect various crops, following the protocol described by Zheng et al. (2019). Agar plugs (8 mm in diameter) of both strain STM-2 and each fungal pathogen were excised from 7-day-old cultures grown on ISP4 and PDA media, respectively. The plugs were placed on opposite sides of a fresh PDA plate, approximately 3 cm apart. The plates were incubated at 28 °C for 5 days. Antagonistic activity was qualitatively assessed using a standardized ordinal inhibition scoring system ranging from 0 (no inhibition) to 3 (strong inhibition). All assays were performed in triplicate to ensure reproducibility.

**Table 1:** List of evaluated phytopathogens and the plants they affect.

Strain	Phytopathogens	Plant affected	Disease
BCRC 30972	*Fusarium proliferatum*	Corn	Sheath rot
BCRC 35046	*Fusarium oxysporum* f. sp. *momordicae*	Bitter squash	Wilt
BCRC 35031	*Neopestalotiopsis* sp.	Wax fruit	Fruit rot
NS-1	*Neopestalotiopsis* sp.	Strawberry	Leaf blight
BCRC 35002	*Neopestalotiopsis* sp.	Guava	Fruit rot
BCRC 35005	*Neopestalotiopsis* sp.	Loquat	Leaf spot
BCRC 35040	*Neopestalotiopsis* sp.	Mango	Leaf spot
BCRC 35178	*Colletotrichum gloeosporioides*	Starfruit	Anthracnose
BCRC 35115	*Fusarium incarnatum-equiseti*(species complex)	Banana	Fruit rot
LC8	*Fusarium* sp.	Strawberry	Wilt

BCRC, Bioresource Collection and Research Center.

### 2.4 Whole genome sequencing, assembly, and annotation

High-molecular-weight DNA was extracted from a 72-h culture of STM-2, grown at 30 °C and 150 rpm in ISP4 broth medium, using the QIAamp PowerFecal Pro DNA Kit (Qiagen). This was followed by size selection with the Short Read Eliminator XS kit (PacBio) to remove DNA fragments smaller than 10 kb. DNA concentration was measured using a Qubit 4.0 fluorometer (Thermo Scientific), and fragment size distribution was assessed with the Qsep 100™ system (BiOptic, Taiwan). Long-read sequencing libraries were constructed using a transposase-based method, which simultaneously cleaves high-molecular-weight genomic DNA and attaches barcoded tags to the cleaved ends. Approximately 1 μg of genomic DNA was processed using the Rapid Barcoding Kit 96 V14 (SQK-RBK114.96, ONT), followed by adapter attachment with a rapid adapter, as per the manufacturer’s instructions. DNA libraries were then purified using Short Fragment Buffer (SFB), which washes fragments larger than 1 kb. Sequencing was performed on a FLO-PRO114M flow cell (R10.4.1) using the ONT PromethION 24 platform.

The raw sequencing data obtained were processed using Dorado’s Super-accurate base calling model (400 bp), and reads with an average quality score above Q10 were retained for downstream analysis. To validate the read length profile, NanoPlot (De Coster et al., 2018) was employed to verify the sequencing results and the raw reads were assembled into primary contigs using Flye (Kolmogorov et al., 2019). Medaka was used to correct and polish the primary contigs. Additionally, the contigs were corrected using homologous sequences extracted from closely related genomes with Homopolish (Huang et al., 2021). The NGS reads were then mapped to the polished contigs and corrected using Pilon (Walker et al., 2014). The quality of the fully polished contigs was assessed using QUAST (Gurevich et al., 2013), while the completeness of the genome was evaluated using BUSCO (Seppey et al., 2019).

The polished genome was first annotated using Prokka (Seemann, 2014) with default settings to predict open reading frames (ORFs) and locate tRNA and rRNA regions. Additionally, gene annotation was performed by aligning the sequences against the Refseq, COG, eggNOG, KOfam, VFDB, CARD, NCyc, dbCAN2, BacMet and PHIBase (Pruitt et al., 2005; Tatusov et al., 2000; Huerta-Cepas et al., 2016; Chen et al., 2005; Jia et al., 2017; Tu et al., 2019; Zhang et al., 2018; Urban et al., 2020; Pal et al., 2013) databases using DIAMOND (Benjamin et al., 2014), HMMER (Mistry et al., 2013), and the database-specific annotators. We then calculated the GC skew and cumulative GC skew using GenSkew (Lu and Salzberg, 2020) and incorporated them with ORF, rRNA, and prophage loci to generate the genome map using Circos (Krzywinski et al., 2009).

To analyze the biosynthetic potential of STM-2, we used antiSMASH version 8.0 (Blin et al., 2025) to identify the number and types of biosynthetic gene clusters for secondary metabolites (BGCs) in the STM-2 genome.

### 2.5 Phylogenomics analysis

*In silico* digital DNA–DNA hybridization (Meier-Kolthoff et al., 2013) was conducted on the GGDC server.^1^ The genome sequence data of STM-2 were uploaded to the Type Strain Genome Server (TYGS) available under https://tygs.dsmz.de, for a whole genome-based analysis (Meier-Kolthoff and Göker, 2019; Meier-Kolthoff et al., 2022). The data provided information regarding synonymy, nomenclature, and associated taxonomic literature is available at https://lpsn.dsmz.de. A phylogenetic tree with whole genomes of STM-2 and 12 closest genomes was generated by TYGS and the tree was visualized in iTOL.^2^ Additionally, whole-genome comparisons between strain STM-2 and the 12 closely related genomes were performed using JSpeciesWS (Richter et al., 2015) to obtain average nucleotide identity (ANIb) values. Average amino acid identity (AAI) analysis was conducted using the EzAAI pipeline (Kim et al., 2021) to obtain AAI values. The tree generated was visualized in iTOL (see text footnote 2). The taxonomic classification of strain STM-2 was confirmed based on the digital DNA–DNA hybridization (dDDH), ANI and AAI values.

### 2.6 Pan-genome, core genome, unique genes, and comparative genomics

To analyze the evolutionary relationships and genomic similarities and uniqueness, we obtained all the genomes of the 12 closely related genomes (*Streptomyces antimycoticus* NBRC12839, *S. melanosporofaciens* DSM40318, *S. antimycoticus subsp. sporoclivatus* NRBRC 100767, *S. mordarskii* JCM 5052, *S. yatensis* JCM 13244, *S. niphimycinicus* 4,503, *S. asiaticus* DSM 41761, *S. iranensis* DSM 41954, *S. asiaticus subsp. ignotus* DSM 41524, *S. rhizosphaericus* DSM 41760, *S. rhizosphaericus* DSM 41769, and *S. rhizosphaericus* DSM 41761) of STM-2 based on the dDDH and ANI values from NCBI.^3^ The genomes were annotated using Prokka (Seemann, 2014). We then conducted a pan-genomics analysis using roary (Page et al., 2015) to determine core genes shared among all the genomes and accessory genes. The pan-genomic analysis was visualized with a heatmap showing gene presence and absence within genomes and a flower plot showing core genes and singletons (unique genes associated with each genome) using Python. We further extracted unique genes associated with STM-2 and produced a bar plot showing the functional categories of the genes using Python.

We compared the in-depth relationship of the most closely related genomes *S. melanosporofaciens* DSM 40318 and *S. antimycoticus* NBRC 12839 and STM-2 by conducting an independent synteny analysis using SyntenyFinder pipeline (Lewin et al., 2025) with slight modifications. The annotated genomes were first used to run orthologous analysis to determine the coordinates of orthologs of all three genomes using OrthoFinder (Emms et al., 2025). Syntenic relationships at the nucleotide level were established based on their respective genomic loci and pairwise synteny plots between the STM-2 genome and each of the two most closely related genomes were generated using SyntenyFinder.

### 2.7 Evolutionary analysis of unique antifungal genes

Unique genes of STM-2 from the roary analysis were further analyzed to identify antifungal genes associated with only STM-2. The FASTA files of the identified unique antifungal genes were then obtained from the annotated genome file of STM-2 using seqkit. We then performed evolutionary analysis to determine the evolutionary trace of these unique antifungal genes. The FASTA files of each genome were blasted to obtain 10 top hits using BLASTp. All FASTA files of each top hit protein were extracted using Python. Mafft was used to align each unique antifungal gene and their top-hits and the phylogenetic trees were generated using IQTree2. The generated trees were cleaned and converted to Newick and visualized using iTOL (see text footnote 2).

### 2.8 Mining of commercially useful enzymes (CUEs) and antifungal genes

The STM-2 genome was mined for CUEs using Python to extract the genes associated with CUEs using the annotated results with RefSeq functional categories. We then generated a bar plot from the results to indicate the number of genes associated with each CUEs.

To understand the genetic basis of the antifungal activity observed in STM-2, we mined and extracted all antifungal genes from the STM-2 genome using biopython. A bar plot showing the functional categories of each and the number of genes was plotted.

### 2.9 *In silico* analysis of proteins of genes encoding for antifungal enzymes produced by STM-2 from enzyme screening

We conducted *in silico* analysis to further confirm the plausible genes identified in the STM-2 genome for encoding antifungal enzymes CMCase, pectinase and xylanase. The genes encoding these proteins were extracted from the annotated file of STM-2. We selected only genes with confirmed RefSeq functions and extracted their protein FASTA using seqkit. The 3D structures of all the proteins were predicted using AlphaFold2 Jupyter pipeline as described earlier by Mirdita et al. (2022) with default settings (model type = auto, num_recycles = 3, relax_max_iterations = 2000, pairing_strategy = greedy, max_msa = auto, num_seeds = 1). The predicted local distance difference test (plDDT) threshold was set as scores ≥ 90 represent very high confidence predictions, while regions with lower scores ≤ 70 may correspond to disordered regions.

## 3 Results

### 3.1 Isolation, characterization and identification of *Streptomyces* strain STM-2

**Figure 2:**
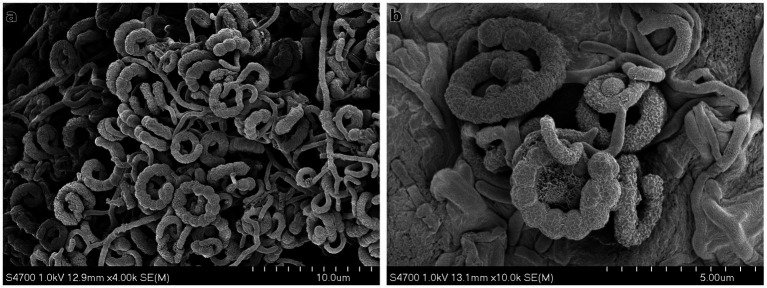
Morphological characteristics of *Streptomyces* strain STM-2 viewed under scanning electron microscopy using **(a)** 4,000x **(b)** 10,000x magnification.

Actinomycete-like colonies appeared on HV agar after 4 weeks of incubation. The strain STM-2 was identified based on its characteristic chalky-white appearance, dry texture, and earthy odor, features typical of *Streptomyces* spp. The colonies were tough and adhered firmly to the agar surface, forming branching aerial mycelia. The isolate was purified by successive streaking and maintained on inorganic salts agar slants and as glycerol stocks at −20 °C. The morphological characteristics of STM-2 were further examined using scanning electron microscopy (SEM). SEM analysis revealed well-developed, spiral spore chains with smooth spore surfaces, consistent with typical *Streptomyces* morphology (Figure 2a). The scanning electron microscopic (SEM) image revealed that the strain had branched mycelia and spiral spores (Figure 2b). These structural features further provided additional confirmation of its identity as a member of the *Streptomyces* genus. The strain STM-2 was incubated on four different International *Streptomyces* Project (ISP) media (ISP2, ISP3, ISP4, and ISP5) and its growth, aerial mycelium, substrate mycelium and sporulation were assessed (Table 2). It was observed that the strain STM-2 could grow on all four ISP media, with *good growth* in ISP2, ISP3 and ISP4 and *moderate growth* in ISP5. The strain showed different substrate and aerial mycelium colorations on the four ISP media. There was *pale yellow* substrate mycelium observed on ISP3 and ISP4, and *moderate yellow* and *light-yellow* substrate mycelium observed on ISP2 and ISP5, respectively. The colours of aerial mycelium of the strain observed were *light gray* on ISP2, *black* on ISP3, *dark gray* on ISP4, and *yellowish white* on ISP5. Sporulation was *good* for all the media except ISP5, where *poor* sporulation was observed. The biochemical and physiological characteristics of *Streptomyces* sp. strain STM-2 are presented in Table 3. The results indicated that the strain could fully utilize glucose, mannitol, raffinose, inositol, fructose, cellobiose, xylose and salicin as carbon sources. Again, the strain could partially utilize sucrose and arabinose as carbon sources. The biochemical tests revealed that the strain could hydrolyze starch and casein, utilize adenine and tyrosine, and was tolerant to 4% NaCl. However, the strain could not reduce nitrate, liquefy gelatin, hydrolyze milk, or peptonize milk and could not utilize urea as a nitrogen source.

**Table 2:** Culture characteristics of strain STM-2 in different ISP media.

Medium	Growth	Substrate mycelium	Aerial mycelium	Sporulation
ISP 2	Good	Moderate yellow	Light gray	Good
ISP 3	Good	Pale yellow	Black	Good
ISP 4	Good	Pale yellow	Dark gray	Good
ISP5	Moderate	Light yellow	Yellowish white	Poor

Good, cell concentration more than 10^8^ cfu ml^−1^; moderate, cell concentration between 10^6^ cfu ml^−1^ and 10^8^ cfu ml^−1^; poor, cell concentration less than 10^6^ cfu ml^−1^.

**Table 3:** Biochemical and physiological characteristics of *Streptomyces* sp. strain STM-2.

Characteristic test result (STM-2)	
Carbon source utilization	
Glucose	+
Mannitol	+
Raffinose	+
Sucrose	+/−
Inositol	+
Arabinose	+/−
Fructose	+
Rhamnose	−
Cellobiose	+
Xylose	+
Salicin	+
Melanin	−
Esculin	−
Biochemical test	
Urease test	−
Milk hydrolysis	−
Milk peptonization	−
Nitrate reductase	−
Hippurate hydrolysis	−
Gelatin liquefaction	−
Tyrosine	+
Xanthine	−
Starch hydrolysis	+
Adenine utilization	+
Casein hydrolysis	+
NaCl tolerance	4%
Hypoxanthine utilization	−

+ = full positive reaction, − = negative reaction, +/− = partial reaction.

### 3.2 Antagonistic activity of strain STM-2

The strain STM-2 showed antagonistic activity against 10 plant pathogens (Table 4). The strain demonstrated strong inhibitory activity against all five tested *Neopestalotiopsis* spp. (*Neopestalotiopsis* sp. BCRC 35031*, Neopestalotiopsis* sp. BCRC NS-1*, Neopestalotiopsis* sp. BCRC 35002*, Neopestalotiopsis* sp. BCRC 35005 and *Neopestalotiopsis* sp. BCRC 35040). The strain STM-2 exhibited moderate antagonistic effects against *Colletotrichum gloeosporioides* BCRC 35178 and all *Fusarium* spp. evaluated.

**Table 4:** Antagonistic activity (inhibition score) of STM-2 against all ten phytopathogens.

Pathogen	Antagonistic effect/score
*Neopestalotiopsis* sp. BCRC 35031	3
*Neopestalotiopsis* sp. NS-1	3
*Neopestalotiopsis* sp. BCRC 35002	3
*Neopestalotiopsis* sp. BCRC35005	3
*Neopestalotiopsis* sp. BCRC 35040	3
*Fusarium proliferatum* BCRC 30972	2
*Fusarium oxysporum* f. sp. *momordicae* BCRC 35046	2
*Colletotrichum gloeosporioides* BCRC 35178	2
*Fusarium incarnatum-equiseti* species complex BCRC 35115	2
*Fusarium* sp.-LC8	2

0, No antagonistic effect; 1, low antagonistic effect; 2, moderate antagonistic effect; 3, high antagonistic effect.

### 3.3 Molecular identification of strain STM-2

The full 16S rRNA gene sequence of strain STM-2 was determined and submitted to the NCBI GenBank database (accession number: PV731399). The 16S rRNA gene sequence analysis for strain STM-2 from the EzBioCloud database showed that the strain exhibited the highest similarity to *Streptomyces antimycoticus* strain NBRC 12839 (99.93%). BLAST results from NCBI further confirmed that STM-2 belongs to the genus *Streptomyces*, exhibiting high sequence similarity ranging from 98.41 to 99.93% with 100 *Streptomyces* strains. The phylogenetic analysis was conducted to determine the evolutionary relationship of the strain with its closely related strains, and the phylogenetic trees have been presented in Figure 1 and Supplementary Figures S1,S2. The neighbor-joining tree, constructed based on 16S rRNA gene sequences, revealed that strain STM-2 clusters within a well-supported clade (bootstrap value: 95%) alongside *Streptomyces melanosporofaciens* strains (DB12, NRRL B-12234, and NBRC 13061), indicating an evolutionary relationship. This close phylogenetic association suggests a high degree of sequence similarity and indicates that STM-2 is most closely related to members of the *S. melanosporofaciens* lineage.

### 3.4 Genomic features and biosynthetic-related gene clusters of strain STM-2

**Figure 3:**
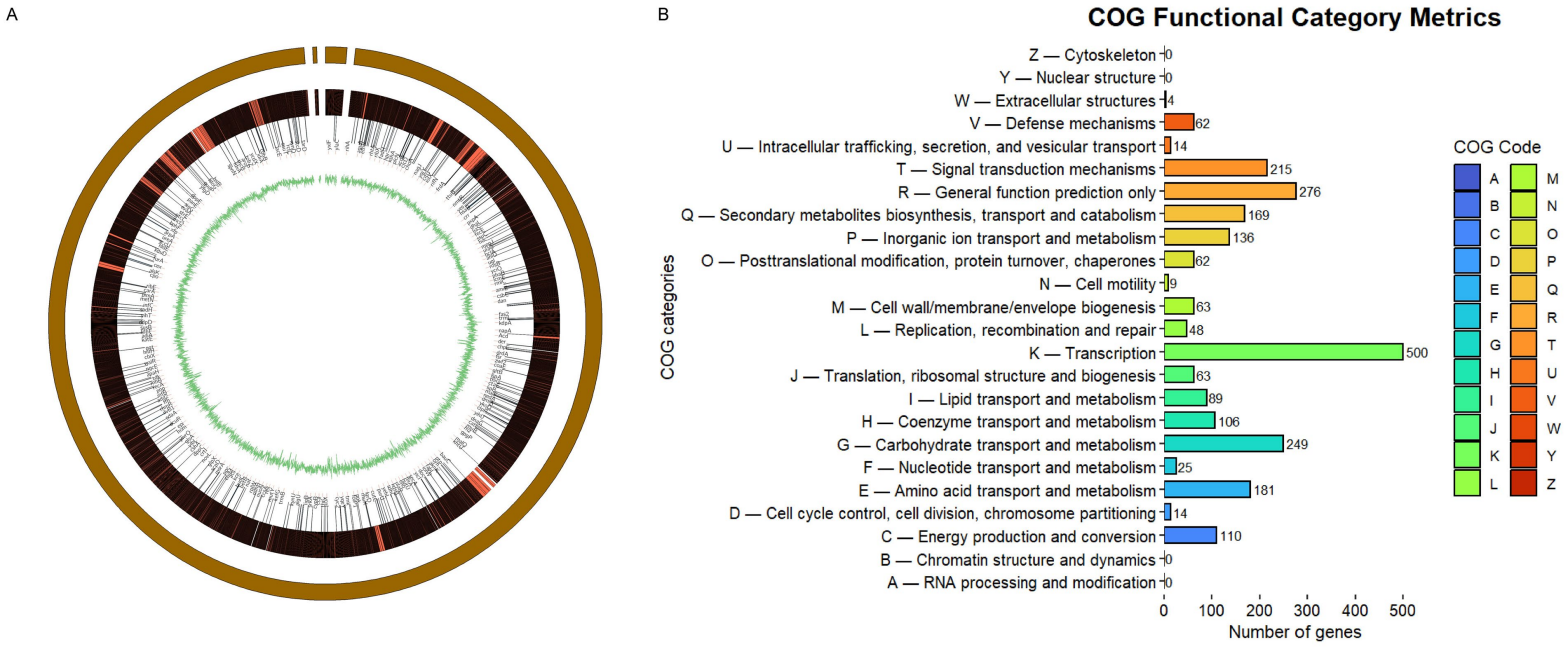
Genomic feature of STM-2. **(A)** Circular genomic map of the complete genome of Steptomyces melanosporofaciens STM-2 generated by Circos, including assembled contigs and genome annotations and GC skew. **(B)** Cluster of Orthologs Gene (COG) database annotation of STM-2 showing functional categories of the STM-2 encoded ORFs.

Strain STM-2 has 3 contigs and a total genome size of 10.8 Mbp (10,784,024 bp); these contain 9,062 protein-coding genes, 84 tRNAs, 18 rRNAs, 139 misc_RNAs, and a G + C content of 71.04%. The circular genome map of STM-2 is shown in Figure 3A, and the classification of the cluster of orthologous genes (COG) is presented in Figure 3B. A total of 2,395 COGs were obtained, with the category with the most COGs being transcription (500). There are 62 COGs for defense mechanisms and 169 COGs for secondary metabolites biosynthesis, transport, and catabolism.

The antiSMASH analysis revealed 52 biosynthetic gene clusters for a wide range of secondary metabolites within the STM-2 genome. All regions for BGCs are found in contig 2 of the genome. The type of BGCs, their compounds, and similarity with known clusters have been presented in Supplementary Table 1. Most antibiotics, anticancer agents, and antifungal compounds that are produced by *Streptomyces* are synthesized via polyketide synthase (PKS) and non-ribosomal peptide synthase (NRPS) pathways. The genome of strain STM-M2 contains 24 regions with clusters related to PKS and NRPS pathways, accounting for 46.2% of all gene clusters. The PKS and NRPS regions include 9 T1 PKS, 1 T2 PKS, 1 T3 PKS, 1 HR-T2PKS, 1 PKS-like, 7 NRPS, and 4 NRPS-like clusters. Additionally, there were 8 terpenes, 2 terpene precursors, 3 RiPP-like, 2 NI-siderophore, 3 betalactone, 1 NRP-metallophore, 1 butyrolactone, 1 lassopeptide, 1 indole, 1 hserlactone, 1 redox-cofactor, 1 ectoine, 1 hydrogen-cyanide, 1 amglyccycl, 1 other cluster identified in the genome. Among the 52 gene clusters encoded in the strain STM-M2 genome, fourteen of the clusters have high similarity confidence with the known gene clusters, indicating that strain STM-M2 has the potential to produce these secondary metabolites. There were 10 clusters with medium similarity confidence and 7 with low similarity confidence to their known gene clusters, suggesting that strain STM-M2 can produce the known metabolites or their structural analogs. The remaining 21 clusters showed no significant matches with known gene clusters, indicating they may represent novel gene clusters.

The digital DNA–DNA hybridization (dDDH) and average nucleotide identity (ANI) values obtained from TYGS results and average amino acid identity valuses obtained from EzAAI analysis were used to determine species delineation among *Streptomyces* spp., with thresholds set at ≥70% for dDDH, ≥95% for ANI and ≥95% for AAI. Comparative analysis of dDDH (Supplementary Table 2), ANI (Supplementary Table 3) and AAI (Supplementary Table 4) values based on the whole genome sequences of strain STM-2 versus the twelve closely related *Streptomyces* strains revealed the highest values of dDDH (d_4_ = 92.9%), ANI (98.79% [88.15%]) and AAI (99.07%) with *Streptomyces melanosporofaciens* DSM 40318. These results confirm that STM-2 belongs to the species *S. melanosporofaciens;* thus, we propose to name this strain *Streptomyces melanosporofaciens* STM-2.

### 3.5 The genome of STM-2 encodes RiPP-like genes

The antiSMASH results revealed a RiPP-like gene cluster in region 2.21 (4,150,528–4,162,900 nt) of the STM-2 genome. RiPPs are peptides synthesized by ribosomes in a template-dependent manner and post-translationally modified by specific enzymes. Based on the MIBiG comparison analysis, it was revealed that the RiPP gene-like gene cluster in region 2.21 was closest (58% similarity) to aromentin-producing BGC (BGC0002277.2) from *Suillus grevillei.* The knownClusterBlast analysis revealed that the RiPP gene cluster exhibits no significant matches to known clusters, indicating that STM-2 may encode a novel RiPP-like peptide. We identified, from the Pfam domains of the RiPP-like cluster (see Supplementary Figure 3), that STM-2 encodes an ATPase and a magnesium-dependent protein, YcaO (DenovoWGSM2-03724). YcaO is an enzyme involved in post-translational modifications that lead to the biosynthesis of a wide range of antibiotics, including bottromycin. YcaO is the core gene in the cluster, predicted from a hypothetical protein encoded by the STM-2 genome annotation. Two AMP-binding enzymes (DenovoWGSM2-03728 and DenovoWGSM2-03729), adh_short (DenovoWGSM2-03719), and FAD_binding_3 (DenovoWGSM2-03721) were identified as additional biosynthetic enzymes.

Another RiPP gene was observed in RiPP-like-T1PKS-hgIE-KS hybrid BGC in region 2.8. The DenovoWGS_M2_00805 gene from the STM-2 genome, a hypothetical protein according to prokka annotation, was identified as an RiPP gene from the Pfam domain analysis of the antiSMASH results. Further NCBI BlastP on the gene revealed that the gene was 100% similar to type A2 lantipeptide (WP_385202518.1) from *Streptomyces* sp. NPDC059455 and indicates the gene could synthesize an RiPP type A2 lantipeptide, which is known to have antimicrobial properties. The region 2.8 cluster also had Peptidase_C39, p450, two Acetyltransf_1, Glyco_hydro_3-Glyco_hydro_3_C, adh_short_C2, Methyltransf_23, UDPGT, Abhydrolase_1, PP-binding, ketoacyl-synt-ketoacyl-synt_c, NMO, Abhydrolase_6, two Methyltransf_11, Esterase_PHB, Aldedh, Cyclase, and Arginase genes as additional biosynthetic genes. The MIBiG comparison showed that the region 2.8 is 59% similar to a PKS BGC (BGC0001273.3) from *Aspergillus ochraceus,* which synthesizes asperlactone, a natural polyketide with antifungal and antibacterial properties. The KnownClusterBlast analysis also showed medium similarity with hexacosalactone A biosynthetic gene cluster (BGC0002497) from *Streptomyces* sp.

### 3.6 STM-2 encodes a wide range of non-ribosomal peptide synthases (NRPS)

Genome mining of STM-2 revealed extensive enrichment of NRPS and NRPS-related biosynthetic gene clusters (BGCs), particularly within contig 2. Multiple NRPS, NRPS-like, and hybrid NRPS clusters were distributed across regions 2.1, 2.4, 2.7, 2.9, 2.23, 2.25, 2.31, 2.37, 2.40, 2.48, 2.49, and 2.50, highlighting a substantial capacity for peptide-based secondary metabolite biosynthesis. The detailed description of these regions, their core biosynthetic genes and regulatory genes have been provided in the Supplementary material. NRPSs are extensive, multimodular enzyme systems found in various bacteria and fungi that synthesize intricate peptides. Distinct from the template-driven process of ribosomal protein synthesis, NRPSs function as enzymatic assembly lines that catalyze peptide formation in a template-independent manner. Peptides resulting from this synthesis demonstrate a broad spectrum of biological functionality, including antimicrobial and anticancer properties (Strieker et al., 2010; Martínez-Núñez and López, 2016). Comparative analysis against the MIBiG database indicated that several clusters share moderate to high similarity with known NRPS BGCs. Region 2.1 was most closely related to the xenematide biosynthetic cluster (72%) but lacked strong KnownClusterBlast matches, suggesting cryptic biosynthetic potential. Region 2.4 exhibited similarity to the atromentin BGC (65%), while region 2.7 showed strong relatedness to the bovienimide A cluster from *Xenorhabdus bovienii* (75%). An NRPS-like-terpene-precursor hybrid cluster was identified in region 2.9, further expanding the predicted chemical space. Region 2.23 showed closest similarity (74%) to the pyreudione biosynthetic cluster from *Pseudomonas fluorescens*, with additional medium similarity to the ochronotic pigment BGC from *Streptomyces avermitilis*. Region 2.25 corresponded most closely to the rhizomide A–C BGC (45%) from *Paraburkholderia rhizoxinica* and exhibited moderate similarity to the skyllamycin A/B cluster. Region 2.31 demonstrated high similarity (86%) to the echoside A BGC from *Streptomyces* sp. LZ35.

Additional NRPS clusters were identified in regions 2.37, 2.40, 2.48, and 2.49, with similarities to the icosalide (74%), choline (53%), pyreudione A (74%), and BD-12 (71%) biosynthetic clusters, respectively. Notably, region 2.48 also showed weak similarity to the glycinocin A cluster, indicating potential novelty. Region 2.50 represented a large hybrid NRP–metallophore–NRPS–T1PKS cluster and displayed very high similarity to the 6-methylsalicylic acid BGC from *Glarea lozoyensis.* Collectively, the abundance, diversity, and partial similarity of STM-2 NRPS clusters to known pathways strongly suggest that this strain harbors significant potential for the biosynthesis of diverse peptide-based natural products, including antimicrobial and bioactive compounds, as well as previously uncharacterized metabolites.

### 3.7 STM-2 encodes a wide range of polyketide synthases (PKS)

The antiSMASH analysis revealed 13 regions in the genomes of STM-2 that encode polyketide synthases encompassing T1PKS, T2PKS, T3PKS, and multiple hybrid architectures with NRPS, terpene, and siderophore biosynthetic systems (detailed description in Supplementary materials). This diversity indicates a broad capacity for polyketide-derived secondary metabolism. Region 2.5 contained a PKS-like-terpene hybrid cluster that was most similar to the naseseazine C, C3 aryl pyrroloindolines BGC (48%) based on MiBG comparison and was closely related to the rustimicin cluster (based on knownClusterBlast), suggesting potential production of antifungal metabolites. Two large T1PKS-NRPS hybrid clusters were identified in regions 2.10 and 2.11, showing high similarity to the hygrocin A/B (91%) and meridamycin (77%) biosynthetic clusters, respectively, based on MiBG comparison analysis results. Region 2.18 represented a T1PKS-NI-siderophore hybrid cluster most closely related to xanthoferrin biosynthesis (56%) and low similarity to the peucechelin BGC, suggesting possible structural divergence. T1PKS cluster in region 2.20 showed high similarity (82%) to the 6-methylsalicylic acid BGC and moderate similarity to the desulfoclethramycin cluster (knownClusterBlast).

An HR-T2PKS hybrid cluster in region 2.22 showed the closest similarity (64%) to the 6-methylsalicylic acid pathway from *Aspergillus terreus* and moderate similarity to a tetraenamide-type BGC. Additional PKS clusters were identified in regions 2.34, 2.39, 2.44, 2.45, 2.46, 2.47, and 2.52. Based on MiBG comparison analysis, these clusters exhibited similarity to BGCs involved in spore pigment production (76%), prolipyrone B (62%), efomycin K (87%), asperlactone (65%), nigericin (88%), niphimycin derivatives (86%), and germicidin (86%), respectively. Polyketide compounds are known to have functional bioactivities, including antibiotics, immunosuppressants, and anticancer therapeutics (Gomes et al., 2013; Tacar et al., 2013; Risdian et al., 2019; Veilumuthu et al., 2024). The PKS and PKS-hybrid repertoire of STM-2 encompasses numerous pathways associated with antimicrobial, antifungal, pigment, and siderophore biosynthesis, and clusters that showed moderate similarity to known BGCs, thus indicating strong potential for the discovery of structurally novel polyketides and hybrid metabolites from this strain.

### 3.8 Comparative genomics and evolutionary analysis reveal unique genes associated with STM-2

**Figure 4:**
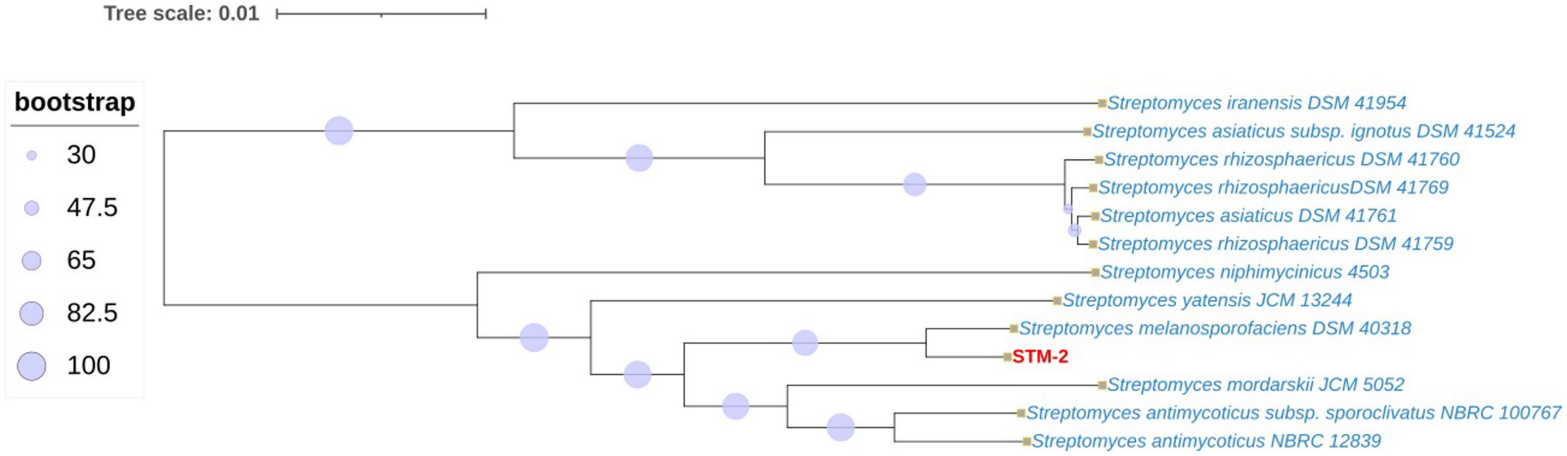
Comparative analysis of strain STM-2 and closely related *Streptomyces* species showing evolutionary relationship. The phylogenetic tree was constructed using the Type Strain Genome Server to analyze the whole genome sequences.

The phylogenetic tree constructed by TYGS based on the whole genome sequences of STM-2 and closely related strains from the DNA–DNA hybridization results showed the highest similarity between STM-2 genome and *Streptomyces melanosporofaciens* DSM40318 genome (Figure 4). The phylogenetic analysis based on whole genome sequences also demonstrated that STM-2 shared a distinct clade with *Streptomyces morfarskii* JCM 5052, *Streptomyces antimycoticus sub*sp. *Sporoclivatus* NBRC 100767 and *Streptomyces antimycoticus* NBRC 12839. Comparative analysis indicated that STM-2 clustered with eight other *Streptomyces* species, suggesting a shared common ancestor. To further validate the phylogeny, we performed roary analysis to determine the core shared genes among the genomes and the presence and absence of genes. When STM-2 was set as the reference genome, the core genome was found to consist of 2,271 CDS, while the pangenome comprised 33,381 CDS (see Supplementary Table 5). Additionally, 11,093 and 20,017 genes were categorized as shell and cloud genes, respectively, forming 90.20% of the pangenome proteome (see Supplementary Table 4).

**Figure 5:**
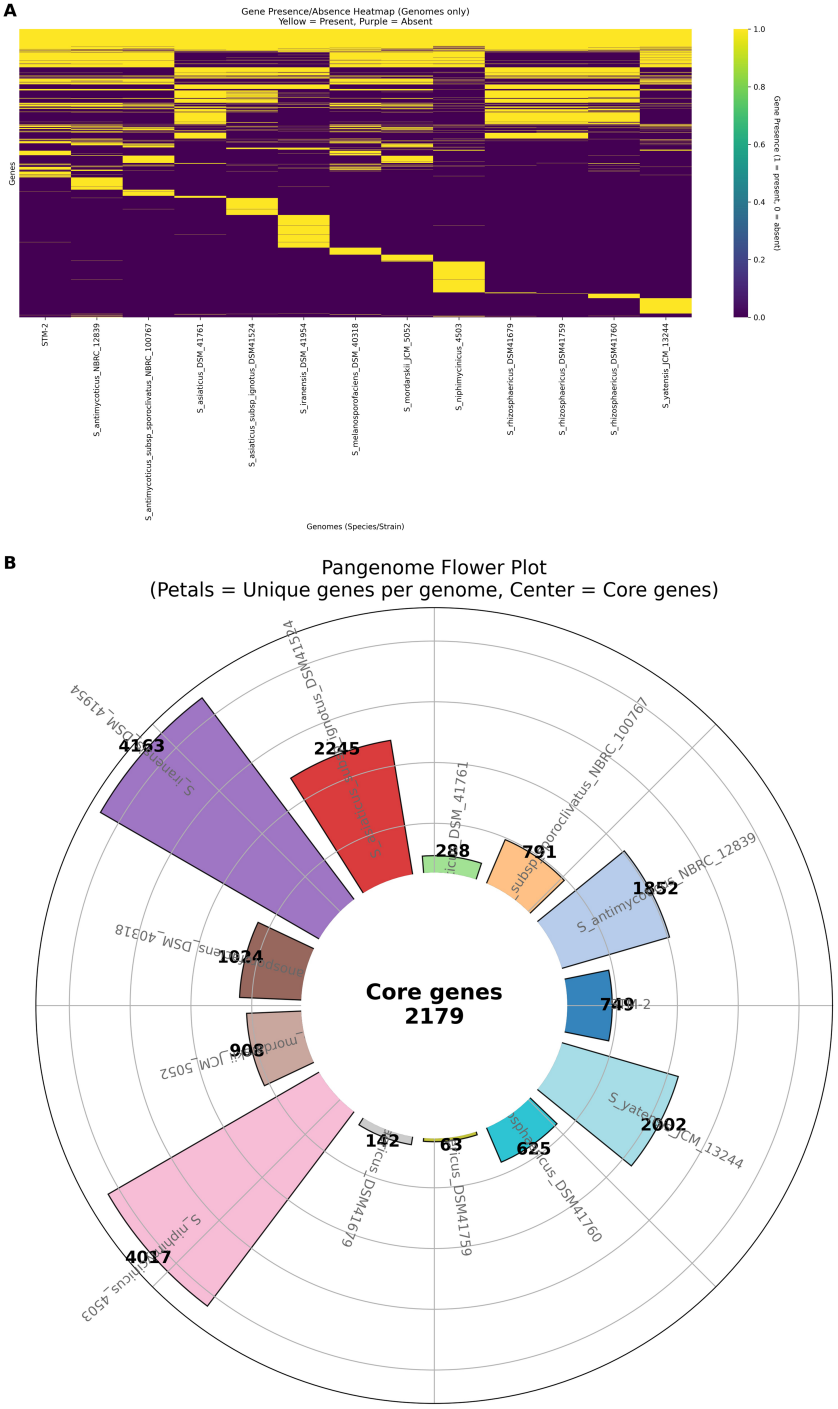
pan-genomic analysis of STM-2 and closest genomes. **(A)** Heatmap showing the gene absence (yellow) and presence (purple) among STM-2 and the 12 closest *Streptomyces* genomes. **(B)** Pan-genome flower plot showing the shared core genes (centre circle) among all the 13 genomes and their respective unique genes (petals).

Figure 5 represents the visualization of the roary analysis results. This roary analysis identified 749 singletons specific to STM-2 in comparison with *S. melanosporofaciens* DSM 40318, *S. antimycoticus sub*sp. *Sporoclivatus* NBRC 100767, *S. antimycoticus* NBRC 12839, *S. morfarskii* JCM 5052, *S. yatensis* JCM 13244, *S. asiaticus* DSM 41761, *S. asiaticus sub*sp. *Ignotus* DSM 41524, *S. iranensis* DSM 41954, *S. niphimycinicus* 4,503, *S. rhizosphaericus* DSM41679, *S. rhizosphaericus* DSM41759, *S. rhizosphaericus* DSM41760 (Figure 5B). There were 605 genes out of the 749 unique genes of STM-2 that were hypothetical proteins; representing novel genes with no known specific function from the annotation databases (see Supplementary Table 6). All other genomes had unique genes specific to them, with the two closest genomes to STM-2, *S. melanosporofaciens* DSM 40318 and *S. antimycoticus* NBRC 12839, having 1,025 and 1852 unique genes, respectively (Figure 5B).

**Figure 6:**
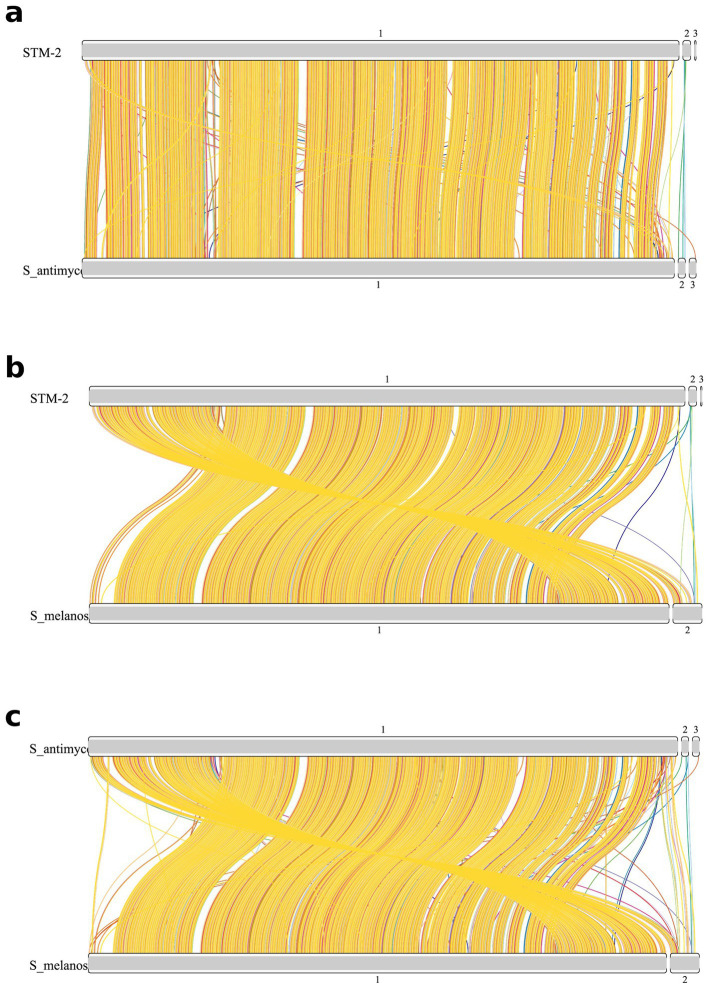
Synteny analysis showing the genome collinearity of **(a)** STM-2/*S. antimycoticus* NBRC12839, **(b)** STM-2/*S. melanosporofaciens* DSM40318 and **(c)** *S. antimycoticus* NBRC12839/*S. melanosporofaciens* DSM40318.

To further compare the in-depth relationship of the most closely related genomes *S. melanosporofaciens* DSM 40318 and *S. antimycoticus* NBRC 12839 and STM-2, we conducted an independent synteny analysis using SyntenyFinder. The synteny plots of the synteny analysis have been presented in Figure 6. The synteny plots 6a, 6b and 6c illustrated the physical positions of shared orthologs within the pairs, enabling the assessment of gene rearrangement and horizontal gene transfer or gene order conservation between these genomes. The absence of an abrupt colour change in the synteny plots indicated collinearity in the gene order of orthologs between the pairs. STM-2 exhibited high similarity with *S. antimycoticus* NBRC 12839 since there was extensive conservation of gene order between the two genomes (Figure 6a). Majority of the syntenic regions were assigned single ancestral linkage group, indicating a highly conserved chromosomal architecture. Few interleaving across the blocks (contigs) were observed. The synteny analysis also revealed high conservation of gene order between STM-2 and *S. melanosporofaciens* DSM 40318 genomes; however, there was a significant number of interleaving suggesting rearrangement or inversions. Over time, genetic differences can accumulate between strains of the same species, which can lead to changes in the gene order between the strains. Therefore, STM-2 might have followed the same evolutionary path compared to *S. melanosporofaciens* DSM 40318 and *S. antimycoticus* NBRC 12839, resulting in the observed collinearity in the synteny plots.

**Figure 7:**
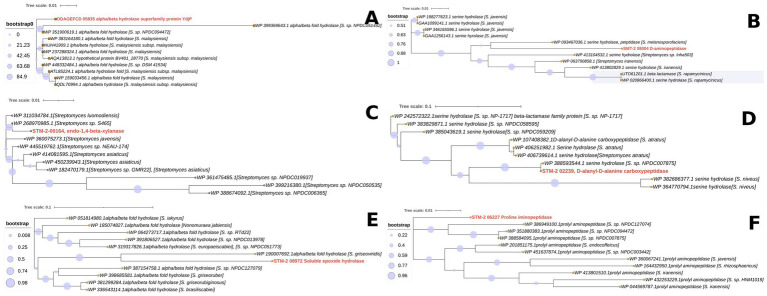
Evolutionary analysis of unique antifungal genes encoded in the STM-2 genome. **(A)** Evolutionary relationship of alpha/beta hydrolase superfamily from STM-2 and top hits enzymes. **(B)** Evolutionary relationship of D-aminopeptidase from STM-2 and top hits enzymes. **(C)** Evolutionary relationship of endo-1-4-beta xylanase from STM-2 and top hits enzymes. **(D)** Evolutionary relationship of D-alanyl-D-alanine carboxypeptidase from STM-2 and top hits enzymes. **(E)** Evolutionary relationship of soluble epoxide hydrolase from STM-2 and top hits enzymes. **(F)** Evolutionary relationship of proline iminopeptidase from STM-2 and top hits enzymes.

Since STM-2 had shown antagonistic activity against phytopathogens, antifungal genes were extracted from the annotation of the unique genes specific to STM-2 and evolutionary analysis was conducted on each unique gene to determine the species from which the genes might have been derived. There were 6 unique antifungal genes specific to STM-2 that were identified from the unique genes of STM-2. The genes were alpha/beta hydrolase superfamily protein Ydjp, D-aminopeptidase, endo-1,4-beta-xylanase, D-alanyl-D-alanine carboxypeptidase, soluble epoxide hydrolase and proline iminopeptidase. Phylogenetic trees exhibiting the evolutionary relationships of each unique CDS and their top 10 Blastp hits have been presented in Figure 7. It was observed from the phylogenetic tree (Figure 7A) that the alpha/beta hydrolase superfamily protein Ydjp from STM-2 was distinct from the other 10 CDS since it did not form any clade with any of the CDS suggesting that the gene might be coding for a novel or distinct protein of peptidase. We observed that the D-aminopeptidase CDS from STM-2 formed the same clade with serine hydrolase peptidase from a *S. melanosporofaciens,* WP 093467036.1 (Figure 7B), which indicates that the *S. melanosporofaciens* DSM 40318 genome had lost the gene. The phylogenetic analysis also revealed that the endo-1,4-beta-xylanase CDS from STM-2 and endo-1,4-beta-xylanase CDS from *Streptomyces* sp. S465 (WP 268970985) belonged to the same clade (Figure 7C), suggesting a strong evolutionary relationship and a horizontal gene transfer (HGT) between the two species. The STM-202239, D-alanyl-D-alanine carboxypeptidase clustered with serine hydrolase group protein from *Streptomyces* sp. NPDC007875 on the same clade (Figure 7D), indicating STM-2 might have gained the gene from this species. It was also observed from the phylogenetic tree (Figure 7E) that the STM-208972 soluble epoxide hydrolase CDS formed the same clade with alpha/beta fold hydrolase from *Streptomyces griseoviridis* (W 19007692.1), suggesting an HGT had occurred between the two genomes. The phylogenetic analysis also revealed that, although, STM-206227 proline iminopeptidase showed sequence similarity with 10 prolyl amino peptidase from *Streptomyces* species, it did not form a clade with any of them. This indicates that the STM-206227 proline iminopeptidase is diverged and more evolved and the gene might have been gained from a distant family.

### 3.9 STM-2 genome encodes commercially useful enzymes

**Figure 8:**
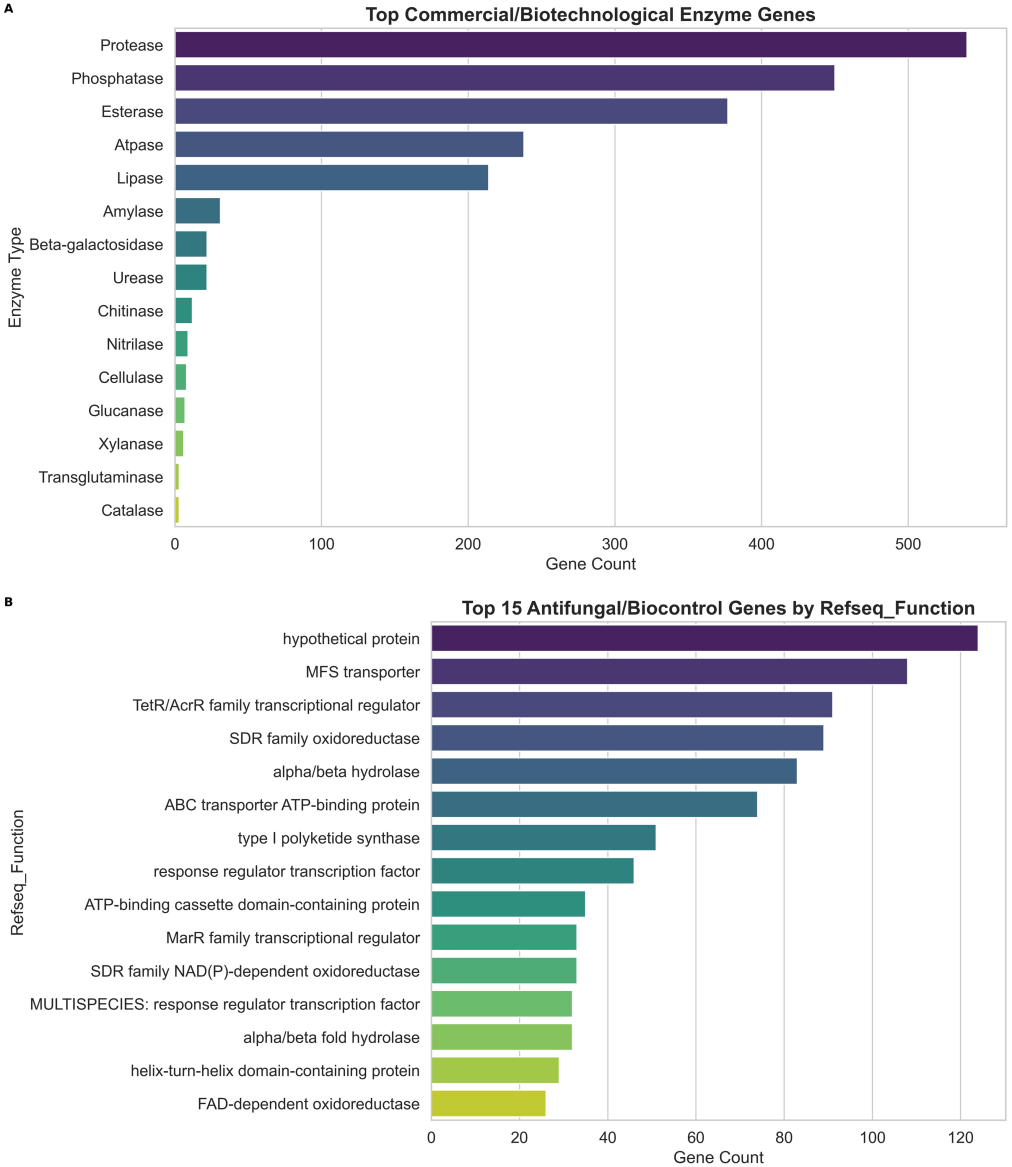
Commercially useful genes encoded in the STM-2 genome. **(A)** Top commercial and biotechnological enzymes genes derived from the STM-2 genome. **(B)** Top antifungal and biocontrol-related genes encoded in the genome of STM-2 based on RefSeq function analysis.

The STM-2 genome was analyzed to find homologs for known commercially useful enzymes (CUEs). This analysis detected a total of 1946 homolog genes encoding CUEs. Figure 8 presents the CUEs genes detected in the STM-2 genome. There were 15 different classes of CUEs detected in the STM-2 genome, with proteases being the most abundant and catalases the least abundant (Figure 8A). Among the enzyme classes identified, chitinases are prominent for their role in fungal cell wall degradation, xylanases for xylan hydrolysis, glucanase for fungal cell wall glucan hydrolysis, and amylases for starch hydrolysis. Collectively, these enzymes represent key genetic determinants underlying the antifungal and biocontrol potential of STM-2. The total number of commercially useful enzymes was analyzed to determine the top 15 antifungal and biocontrol–related genes based on RefSeq functional annotation (Figure 8B). Most of the genes were hypothetical proteins, however, a significant number of genes were involved in type I polyketide synthase.

### 3.10 Analysis of extracellular enzymes and *in-silico* analysis of putative genes encoding these enzymes

**Figure 9:**
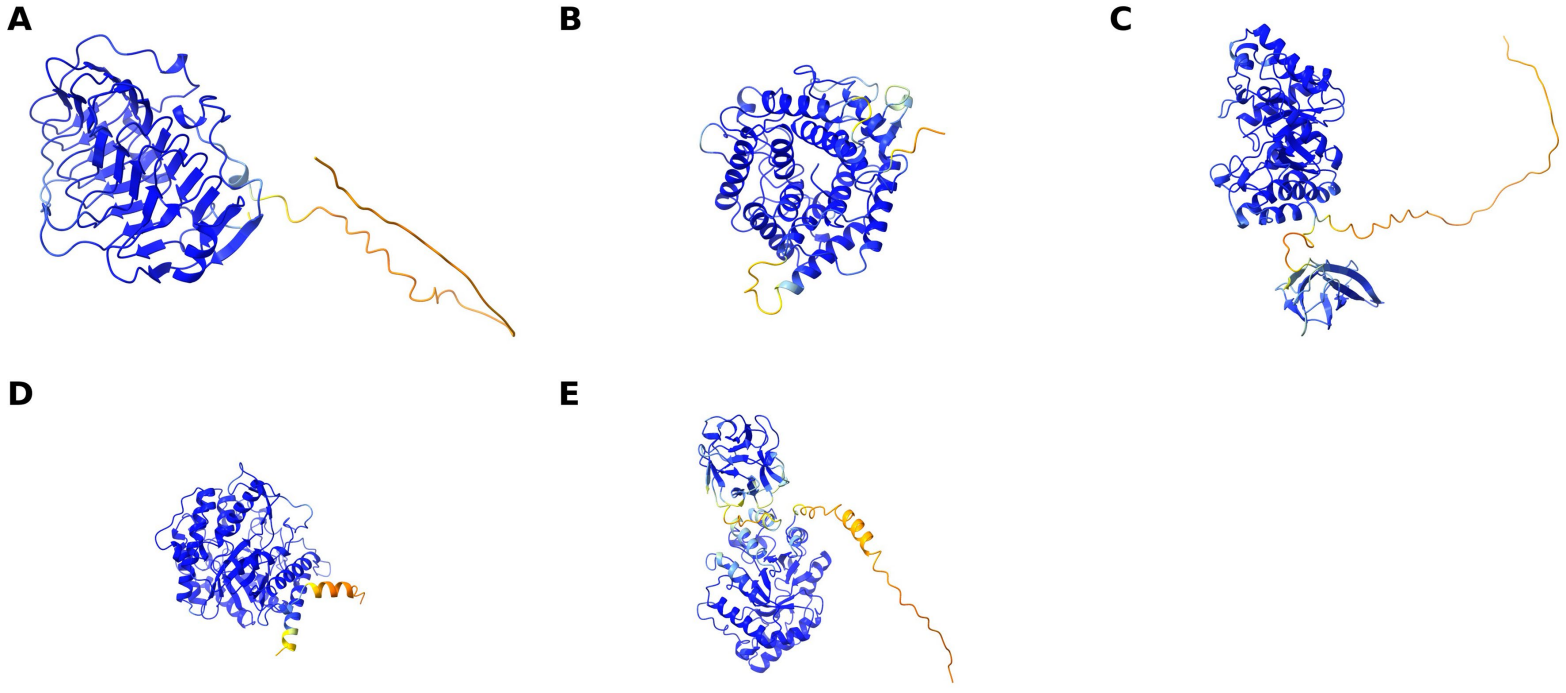
Alphafold2-predicted three-dimensional protein structures of antifungal enzymes from STM-2 enzyme screening. **(A)** predicted 3D-fold of STM-2_01008Pectatetrisaccharidelyase, **(B)** predicted 3D-fold of STM-2_04488Endoglucanase, **(C)** predicted 3D-fold of STM-2_07670xylanase, **(D)** predicted 3D-fold of STM-2_08251Betaglucosidase, **(E)** predicted 3D-fold of STM-2_00164Endo-1,4-beta-xylanase. Different colours of the parts of the structure represent confidence levels across the amino-acid sequence, with higher scores indicating greater reliability of the predicted structure. Blue (>90)-very high, Cyan (80)-confident, Green (70)-OK, Yellow (60)- low, Red (<50)- very low.

**Figure 10:**
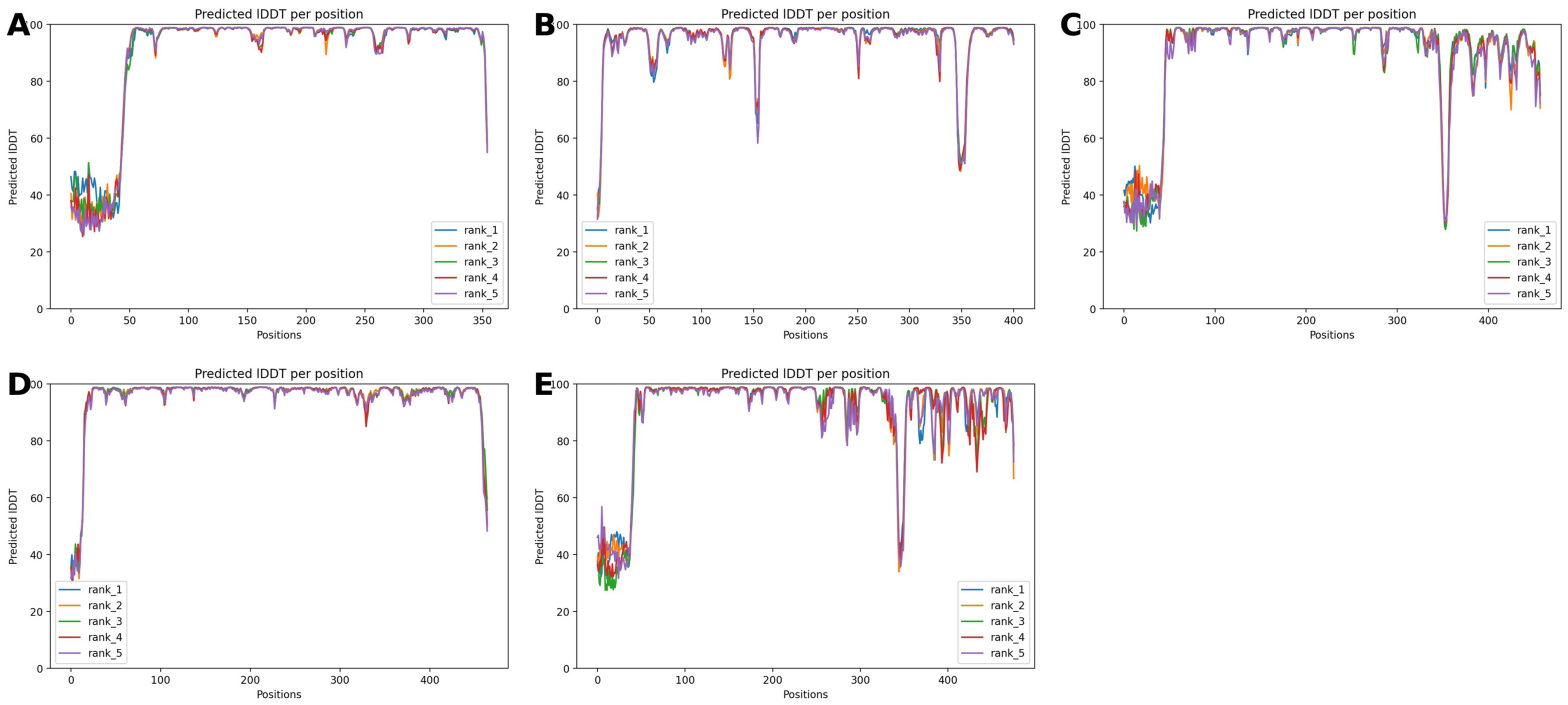
Alphafold2-predicted three-dimensional structure per-residue confidence (pLDDT) plots of antifungal enzymes from STM-2 enzyme screening. **(A)** pLDDT plot of STM-2_01008Pectatetrisaccharidelyase, **(B)** pLDDT plot of STM-2_04488Endoglucanase, **(C)** pLDDT plot of STM-2_07670xylanase, **(D)** pLDDT plot of STM-2_08251Betaglucosidase, **(E)** pLDDT plot of STM-2_00164Endo-1,4-beta-xylanase. The pLDDT plots illustrate the confidence level across the amino-acid sequence, with higher scores indicating greater reliability of the predicted structure. Positions with pLDDT scores ≥ 90 represent very high confidence predictions, while regions with lower scores ≤ 70 may correspond to disordered regions.

The enzyme screening revealed that *Streptomyces* sp. strain STM-2 can produce amylase, protease, carboxymethyl cellulase, pectinase and xylanase (Table 5). There were very strong positive reactions observed for pectinase and xylanase. To further evaluate the antifungal enzymes, the candidate genes for CMCase, pectinase and xylanase were extracted from the genome annotation file and the results have been presented in Supplementary Table 7. There was more than one copy of each enzyme observed in the genome. CMCase had 8 copies, Xylanase and pectinase had 6 copies each. To further validate the functions of the identified genes, AlphaFold2 was used to predict protein structures using protein sequences extracted from the FASTA file of the STM-2 genome, with only five genes having original protein labels. The Alphafold2 structures of the five enzymes are presented in Figure 9. All predicted folds exhibited very high confidence (>90%), with most of the parts as shown in Figure 10, supporting the structural integrity of the predicted proteins and reinforcing their functional plausibility as observed.

**Table 5:** Enzyme screening results for *Streptomyces* sp. Strain STM-2.

Enzyme screened	Result
Amylase	+
Protease	+
Lipase	−
Chitinase	−
CMCase	+
Pectinase	++
Xylanase	++
Ligninase	NG

CMCase, carboxymethyl cellulase; NG, no strain growth; + = positive reaction; ++ = very strong positive reaction; − = negative reaction.

## 4 Discussion

The isolation and characterization of *Streptomyces melanosporofaciens* strain STM-2 revealed several features consistent with the genus *Streptomyces*. Morphologically, strain STM-2 formed chalky-white colonies with branching aerial mycelia and spiral spore chains, aligning with documented characteristics of *S. melanosporofaciens* (Aoyagi et al., 1992) and *S. antimycoticus* (Komaki and Tamura, 2020). SEM analysis confirmed the presence of wrinkled spiral spores, further supporting its taxonomic placement within the genus *Streptomyces* (Idris et al., 2017). The growth of strain STM-2 across various ISP media highlighted its robust adaptability and sporulation capacity, especially on ISP2, ISP3, and ISP4. The variations in aerial and substrate mycelium coloration are typical of *Streptomyces* spp. and are influenced by nutrient availability and environmental conditions (Barka et al., 2016). Strain STM-2 demonstrated pronounced growth and sporulation on 95 g of rice grain with 5 g of rice bran (Results not shown). This shows that strain STM-2 is metabolically active on diverse nutrients, and low-cost media like rice can be utilized for mass solid fermentation and metabolite production. The current study revealed that strain STM-2 exhibited broad carbon source utilization and enzyme production profiles during the biochemical tests. The ability to hydrolyze starch, casein, and utilize adenine and tyrosine agrees with the biochemical capabilities of *S. antimycoticus* strains (Komaki and Tamura, 2020) and *S. melanosporofaciens* described in the literature (Aoyagi et al., 1992). Furthermore, strain STM-2 produced high levels of pectinase and xylanase, enzymes with significant agro-industrial relevance, suggesting potential applications in plant biomass degradation and biocontrol strategies.

Several studies have reported the utilization of antagonistic *Streptomyces* species for plant disease control. For instance, Zhong et al. (2023) earlier reported on utilizing *S. olivoreticuli* ZZ-21 against tobacco target spot, caused by *Rhizoctonia solani,* while *Streptomyces* sp. Y1-14 has proven effective against banana *Fusarium* wilt (Cao et al., 2022). Additionally, previous studies have reported on *Streptomyces* species producing diverse secondary metabolites that have been formulated as pesticides for agricultural applications including avermectin (Li et al., 2010), jinggangmycin and zhongshengmycin (Dong et al., 2023). In this study, *Streptomyces* sp. strain STM-2 showed antagonistic effects against 10 phytopathogens that were co-cultured with the strain. This antagonistic effect is consistent with earlier reports highlighting the biocontrol potential of *Streptomyces melanonosporofaciens*, a species known for producing diverse secondary metabolites with antifungal properties, including geldanamycin and elaiophylin (Beauséjour et al., 2003; Ding et al., 2023). The inhibition of growth of the 10 phytopathogens in this study indicates that strain STM-2 may produce diffusible bioactive compounds that interfere with pathogen growth mechanisms such as cell wall disruptions, signaling pathways or competition for nutrients and space. The ability of strain STM-2 to produce extracellular hydrolytic enzymes, including amylase, protease, cellulase (CMCase), pectinase, and xylanase could also be a factor for the antagonistic activity. These enzymes are likely to contribute to its antagonistic potential by degrading structural polysaccharides and glycoproteins that make up the cell walls of phytopathogenic fungi. Enzymatic lysis weakens the pathogen’s physical barrier and can enhance the diffusion and efficacy of antifungal metabolites (Palaniyandi et al., 2013; El-Tarabily et al., 2021). Some strains of *Streptomyces melanosporofaciens* have been successfully applied as biological control agents in agricultural systems. For instance, Beauséjour et al. (2003) earlier reported that *S. melanosporofaciens* strain EF-76 effectively suppressed common scab in potato and demonstrated protective ability under both field and greenhouse conditions. This was later confirmed by Clermont et al. (2010), who reported that *Streptomyces melanosporofaciens* EF-76 produces geldanamycin, a compound with antifungal properties effective against various gram-positive bacteria and fungi. Similarly, *S. melanosporofaciens* strain X216 reduced clubroot disease severity in *Brassica napus* and positively altered rhizosphere microbial diversity, highlighting its ecological compatibility and biocontrol efficacy (Ding et al., 2023).

The integration of whole-genome sequencing (WGS) and detailed bioinformatics analysis is essential for understanding the molecular basis of the antagonistic activity observed in STM-2. Specifically, it allows for the identification of key genes underlying the synthesis of specific compounds and useful antimicrobials. While traditional *in vitro* assays confirm phenotypic growth inhibition against plant pathogens, they often fail to capture the full biosynthetic potential of a strain, as many biosynthetic gene clusters (BGCs) remain “cryptic” or “silent” under standard laboratory conditions (Popov et al., 2025). WGS allows for the comprehensive identification of these hidden genomic determinants, providing a genomic landscape that explains the dual-action biocontrol mechanisms: secondary metabolite production and enzymatic secretion that drive a strain’s efficacy (Veilumuthu et al., 2024). The WGS aided in the identification and classification of STM-2, a comprehensive analysis of the genome and bioprospecting of potential secondary metabolite gene clusters and CUEs genes. The taxonomic classification of the STM-2 was established through Average Nucleotide Identity (ANI) and digital DNA–DNA hybridization (dDDH), using standard species-boundary thresholds of ≥95% and ≥70%, respectively (Xu et al., 2024). Phylogenomic analysis of the WGS of STM-2 revealed *S. melanosporofaciens* as the most closely related strain, with a dDDH of 92.4% and ANI of 98.79%. These findings supported the 16S rRNA phylogenetic tree, which placed STM-2 in the same clade as *S. melanosporofaciens* strain DB12.

The current study revealed several aspects of *S. melanosporofaciens* that have been rarely studied until now. To further facilitate the study and use of STM-2, a thorough investigation of the genes responsible for producing natural compounds and CUEs is required. The genome annotation and biosynthetic analysis of the STM-2 genome revealed large repertoires of biosynthetic genes and commercially useful enzymes. AntiSMASH BGC analysis revealed that STM-2 has the potential to produce a wide range of polyketides, NRPS, RiPP-like genes, and TPS. *Streptomyces* species produce a diverse range of secondary metabolites, primarily through the biosynthesis of polyketides, nucleosides, peptides, and hydrolytic enzymes. These secondary metabolites and hydrolytic enzymes are capable of inhibiting the growth of pathogens or even killing pathogens. Additionally, *Streptomyces* generates various bioactive compounds with antimicrobial properties, including enzymes, organic acids, amino acids, immunomodulators, and vitamins (Dong et al., 2023; Li et al., 2010; Sharma et al., 2022). Specifically, STM-2 has the potential of producing YcaO, an RiPP which is involved in synthesis of bottromycin. Bottromycin is known to have antimicrobial activity against wide range of pathogens, and it is known to be synthesized by *Streptomyces bottropensis* (Gomez-Escribano et al., 2012), *Streptomyces* sp. BC16019 (Hou et al., 2012). The similarity scores of the RiPP-like BGC in region 2.21 of STM-2 genome was extremely low, indicating that STM-2 has the potential to produce a novel RiPP. Another RiPP gene observed in STM-2 genome was revealed to be 100% similar to type A2 lantipeptide, suggesting that STM-2 has the potential of producing a type A2 lantipeptide, a class of compounds known for their antimicrobial properties. AntiSMASH analysis also revealed that STM-2 has potential to produce PKS compounds such as efomycin, rustmicin, and niphimycin which are known to have antifungal and antibacterial activity. Klassen et al. (2019) recognized that the high antifungal activity of the termite-associated *Streptomyces* sp. M56 was partly attributed to the production of the antimicrobial compound efomycin. Again, a study conducted by Mandala et al. (1998) revealed the antifungal potent of rustmicin. Kim et al. (2012) also isolated niphimycin, with broad-spectrum antimicrobial activities from *Streptomyces* sp. These results support the potential for STM-2 inhibiting growth of the phytopathogens used in this study. STM-2 has the potential to produce PKS hygrocins which have shown antitumor activity on breast cancer MDA-MB-431 cells (Lu et al., 2013) indicating that STM-2 can also be utilized as antitumor producing agent in pharmaceuticals. Genome mining analysis also revealed the potential of STM-2 synthesising wide range such as atromentin, xenematide, pyreudione, glycinocin from its NRPS biosynthetic gene cluster regions. These compounds are known to have antibacterial activities as described earlier by Hamers et al. (2020), such as atromentin against *E. aerognes* (Lang et al., 2008); xenematide against *E. coli, B. subtilis* and *Staphylococcus lentus* (Klapper et al., 2016); and pyreudione protecting producers against amoebal predation. Together, these findings support the potential of STM-2 as a promising source of antibacterial metabolites.

Beyond mere identification, comparative genomics and pangenomic analysis are critical for determining a strain’s uniqueness (Popov et al., 2025). By delineating the core genome from the accessory genome, researchers can pinpoint unique genes often associated with specialized antifungal functions or environmental stress responses that are absent in closely related but less active strains (Xu et al., 2024). In the current study, gene family clustering indicated the sharing of 2,271 genes, with STM-2 having 749 unique genes, of which six were known antifungal genes. The evolutionary analysis of these unique antifungal genes revealed that the genes were gained from different genera and species. These unique genomic determinants often result from evolutionary forces like horizontal gene transfer, allowing the strain to adapt to specific ecological niches, such as the rhizosphere (Xu et al., 2024). Again, syntenic analysis revealed poor genomic alignment between strain STM-2 and *S. melanosprofaciens* DMS40318, contrasted by a comparatively higher level of synteny with *S. antimycoticus* NBRC12839, reflecting numerous insertions, deletions, inversions, and translocations across the genome. The AAI analysis revealed that the proteome coverage between STM-2 and *S. melanosprofaciens* DMS40318 was less 90% which supports the synteny analysis observation suggesting divergence and loss and gain of genes. A similar result was reported by Xu et al. (2024), where syntenic analysis revealed poor genomic alignment between *S. luteireticuli* strain ASG80 and strains JCM4788, JCM4087, and CMAA1322. These findings imply that strain STM-2 possesses distinctive characteristics and genomic determinants, and this may explain its biocontrol mechanisms, which significantly differ from those observed in other *Streptomyces* strains.

Enzymatic lysis weakens the pathogen’s physical barrier and can enhance the diffusion and efficacy of antifungal metabolites (Palaniyandi et al., 2013; El-Tarabily et al., 2021). This current research revealed that STM-2 produces extracellular hydrolytic enzymes such as CMCase, pectinase, and xylanase and the genomic analysis revealed several genes related to the synthesis of these extracellular hydrolytic enzymes. To bridge the gap between genomic potential and functional enzymes of STM-2, AlphaFold2 was employed to predict the three-dimensional structures of unique antifungal proteins. This neural network-based approach incorporates physical and biological knowledge to achieve near-experimental atomic accuracy (Jumper et al., 2021). The reliability of these predictions is quantified by the predicted local-distance difference test (pLDDT), where scores above 70–90 indicate high confidence in the local fold and side-chain orientations (Jumper et al., 2021). In this study, the high pLDDT scores obtained for predicted antifungal enzymes provide a high-confidence basis for understanding the structural determinants of the STM-2’s unique antifungal activity, potentially identifying novel binding sites or catalytic domains (Kim et al., 2025).

## 5 Conclusion

The *Streptomyces melanosporofaciens* strain STM-2 was isolated from soil sample in Chiayi County, Taiwan. The strain showed features and characteristics that place it in the *Streptomyces* genus. The antagonistic activity of strain STM-2 against phytopathogens and its ability to synthesize CUEs demonstrate its functional versatility. The transition from *in vitro* observation to whole-genome sequence annotation was pivotal, as it allowed for the comprehensive mapping of STM-2’s metabolic potential and revealed expansive repertoires of biosynthetic gene clusters (BGCs), including polyketides, non-ribosomal peptide synthetases (NRPS), and RiPP-like genes. Comparative pangenomic analysis, supported by high-resolution Average Nucleotide Identity (ANI) and digital DNA–DNA hybridization (dDDH), successfully delineated the core genome from the accessory genome, revealing a significant set of unique genes associated specifically with STM-2’s specialized antifungal capabilities. Furthermore, the application of AlphaFold2 to model the 3D structures of these unique antifungal proteins bridged the gap between sequence and function, with very high pLDDT scores providing high-confidence structural evidence for their specialized enzymatic roles. Collectively, these results underscore the power of integrating genome mining, comparative genomics, and artificial intelligence to move beyond descriptive biology toward a precise, molecular understanding of a strain’s biocontrol toolkit. Ultimately, this study positions the characterized *Streptomyces melanosporofaciens* STM-2 as a highly promising and sustainable candidate for biological control formulations in agricultural and pharmaceutical biotechnology.

## Data Availability

The original contributions presented in the study are publicly available. This data can be found at the National Center for Biotechnology Information (NCBI) using accession number PRJNA1402141.

## Footnotes

1
https://ggdc.dsmz.de/

2
https://itol.embl.de/tree/

3 ncbi.nlm.nih.gov/datasets/genome/
